# Reviewed article: Guerreiro BA, Lara LS, Gialain IO, Silva CSV, Volpato LER. Analysis of the profile of patients admitted to an orofacial cleft rehabilitation service: an observational study, Cuiabá, 2005-2023. Epidemiol Serv Saude. 2025:34;e20240926

**DOI:** 10.1590/S2237-96222025v34e20240926.c

**Published:** 2025-10-24

**Authors:** Fernanda Campos de Almeida Carrer

**Affiliations:** 1 Universidade de São Paulo Faculdade de Odontologia, São Paulo, SP, Brazil

## First round comments

Prezados autores,

Congratulo os autores pela escolha do tema, considero, após leitura atenta do manuscrito e dos pareceres muito bem elaborados dos revisores, que o manuscrito pode ser adequado se os autores considerarem as inúmeras sugestões dos pareceres em anexo. Peço especial atenção aos capítulos de método, resultado e discussão, pois “há necessidade de aprimorar a discussão e trazer os resultados encontrados na pesquisa em contraste com a literatura nacional e/ou internacional que encontraram informações semelhantes ou divergentes”, como afirma uma das revisoras. Ademais, as conclusões precisam encontrar lastro nos resultados, cuidado com extrapolações que nem o método, nem os resultados permitem.

Apenas na próxima versão será possível, de fato, verificar se o manuscrito poderá ser considerado para publicação, mas observamos que há um potencial, se o mesmo for completamente adequado.

Recommendation: Major revision

